# Whole genome re-sequencing of sweet cherry (*Prunus avium* L.) yields insights into genomic diversity of a fruit species

**DOI:** 10.1038/s41438-020-0281-9

**Published:** 2020-05-01

**Authors:** Aliki Xanthopoulou, Maria Manioudaki, Christos Bazakos, Christos Kissoudis, Anna-Maria Farsakoglou, Evangelos Karagiannis, Michail Michailidis, Chrysanthi Polychroniadou, Antonios Zambounis, Konstantinos Kazantzis, Athanasios Tsaftaris, Panagiotis Madesis, Filippos Aravanopoulos, Athanassios Molassiotis, Ioannis Ganopoulos

**Affiliations:** 1 Laboratory of Pomology, Department of Agriculture, Aristotle University of Thessaloniki, 54124 Thessaloniki, Greece; 2 Institute of Plant Breeding and Genetic Resources, ELGO-DEMETER. Thermi, Thessaloniki, 570001 Greece; 3 Perrotis College, American Farm School, Thessaloniki, GR-57001 Greece; 4 Laboratory of Forest Genetics & Tree Breeding, Faculty of Agriculture, Forestry & Environmental Science, Aristotle University of Thessaloniki, Thessaloniki, Greece; 5 Institute of Plant Breeding and Genetic Resources, ELGO-DEMETER. Department of Deciduous Fruit Growing, Naoussa, 59035 Greece; 6 Institute of Applied Biosciences, CERTH, Thermi, Thessaloniki, 570 01 Greece

**Keywords:** Comparative genomics, Next-generation sequencing

## Abstract

Sweet cherries, *Prunus avium* L. (*Rosaceae*), are gaining importance due to their perenniallity and nutritional attributes beneficial for human health. Interestingly, sweet cherry cultivars exhibit a wide range of phenotypic diversity in important agronomic traits, such as flowering time and defense reactions against pathogens. In this study, whole-genome resequencing (WGRS) was employed to characterize genetic variation, population structure and allelic variants in a panel of 20 sweet cherry and one wild cherry genotypes, embodying the majority of cultivated Greek germplasm and a representative of a local wild cherry elite phenotype. The 21 genotypes were sequenced in an average depth of coverage of 33.91×. and effective mapping depth, to the genomic reference sequence of ‘Satonishiki’ cultivar, between 22.21× to 36.62×. Discriminant analysis of principal components (DAPC) with SNPs revealed two clusters of genotypes. There was a rapid linkage disequilibrium decay, as the majority of SNP pairs with *r*^2^ in near complete disequilibrium (>0.8) were found at physical distances less than 10 kb. Functional analysis of the variants showed that the genomic ratio of non-synonymous/synonymous (dN/dS) changes was 1.78. The higher dN frequency in the Greek cohort of sweet cherry could be the result of artificial selection pressure imposed by breeding, in combination with the vegetative propagation of domesticated cultivars through grafting. The majority of SNPs with high impact (e.g., stop codon gaining, frameshift), were identified in genes involved in flowering time, dormancy and defense reactions against pathogens, providing promising resources for future breeding programs. Our study has established the foundation for further large scale characterization of sweet cherry germplasm, enabling breeders to incorporate diverse germplasm and allelic variants to fine tune flowering and maturity time and disease resistance in sweet cherry cultivars.

## Introduction

*Prunus avium* L. (*Rosaceae*), is a fruit crop with growing agronomic and economical importance. For instance, in the past decade, production acreage of sweet cherries in Greece has been increased by 4000 ha, representing more than a 50% increase, while production has also increased by roughly the same percentage to an average of 73,000 tons per year in 2016 (FAOSTAT, 2016) (http://www.fao.org/faostat/en/?#data/QC/visualize). Sweet cherry consumption has potential preventative benefits against (among others) Alzheimer’s, cancer, and inflammation related diseases^1^. Due to the economic importance of sweet cherry, its potential benefit for human health and its advancing position in Greek agriculture, it is vital to provide research support that would maintain global competitiveness for local growers^2^.

Sweet cherries exhibit important phenotypic variation in fruit size, shape, color, sugar content, flowering time and other agronomic traits such as defense reactions against pathogens^3^. The reason for the development of genetic variability in sweet cherry cultivars which originate from Black and Caspian Seas is their adaptation in the context of spreading. The deeper study and characterization of sweet cherry genetic diversity as well as the identification of genes controlling traits of interest will be a key factor for sweet cherry breeding. Greece contains high rates of genetic diversity, although many of local traditional landraces have been lost over years of evolution^2^. Moreover, in various other countries, a narrow genetic bottleneck has been observed, in modern cultivars^4^. In Greece, various studies of molecular diversity, of modern cherry varieties, have been conducted using SSR markers, revealing their extensive genetic basis, a valuable finding that has been extensively used in breeding programs^2,5^. Genomic characterization of sweet cherry germplasm, has been enriched with tools, such as the sweet and sour cherry 6 K array^6^. Whole Genome Re-Sequencing and other such techniques are the springboard for the discovery of allelic and genomic richness.

Identifying allelic variation has gone a step further with the introduction of re-sequencing strategies. Deeper sequencing of genotypes of numerous species has been achieved with the use of high-throughput Next-Generation Sequencing technologies for genome-wide analysis. The genetic background of various fruit-tree species, including citrus^7^, plum^8^, mei^9^, and peach^10^ has been studied with the recently developed, Whole-genome re-sequencing (WGRS) technique. In order to better understand the genetic basis of plant variation and to incorporate this knowledge into breeding programs, the exploitation of valuable sequences continuously provided in public databases, is of the utmost importance.

The genetic basis of phenotypic diversity has become even more enlightened by the use of genomic resources, such as the development of single nucleotide polymorphism (SNP) maps of genomes. Furthermore, a variety of polymorphisms in underlying loci has been recognized^11^. Perennials, including fruit trees, have been studied to a much smaller scale compared to annual plants. In order to evolve breeding and ensure global food security, the exploitation of genomic resources and phenotypic diversity of perennial taxa is of paramount importance^12^.

The recent release of the first version of sweet cherry genome assembly of ‘Satonishiki’ cultivar by Shirasawa et al.^13^ in conjunction with the release of chloroplast and mitochondrial genome sequences^14,15^ have enabled new discoveries, including the identification of the SI locus^16^.

In this article, we present analyses of whole genome re-sequencing of 20 sweet-cherry cultivars and one wild-cherry genotype. The 20 Greek genotypes span the traditional range of sweet cherry cultivation and represent the majority of variation (>95%) in Greece^2^ while this collection is complemented by the presence of an elite local wild cherry genotype. The sequence analysis focused on genomic regions associated with propitious variation, such as deletions, substitutions and duplications, providing the first comprehensive catalog of molecular variation in this species, which could be helpful for explaining divergence/similarity among different variants.

## Materials and methods

### Plant material

Twenty cultivated sweet-cherry genotypes were selected (Supplementary File 1) from the Greek Fruit Gene Bank collection in Naousa (Institute of Plant Breeding and Genetic Resources-H.A.O. ELGO DEMETER), in Greece, to represent the total diversity of Greek sweet-cherry cultivars^2^. The elite wild cherry genotype was obtained from the Wild Cherry Gene Bank, located in the Xyloupolis Forest Nursery of the Hellenic Forest Service in Greece. The 20 sweet cherry accessions are traditional Greek cultivars, whereas the wild cherry one, was used as an outgroup. We predefined four groups as ‘Breeding line’, ‘Landrace’, ‘Modern cultivar’ and ‘Wild’ (Supplementary File 1). The morpho-physiological characterization of these twenty sweet cherry cultivars, has been described previously^17^. Genomic DNA was isolated from a pool of young leaf samples of five plants per genotype using NucleoSpin Plant II kit (Macherey-Nagel).

### Library construction and whole genome re-sequencing

Isolated DNA was fragmented with Bioruptor (ThermoFisher Scientific, Waltham, MA, USA) to generate of approximately 300 bp library insert size. Quantity and quality control of the libraries were carried out with Qubit dsDNA HS Assay kit (ThermoFisher Scientific) and Agilent 2100 Bioanalyzer System (Agilent Technologies, Santa Clara, CA, USA), respectively. High-quality DNA libraries were sequenced with the BGISEQ-500 platform (BGI-Tianjin) with read lengths of 100 bp.

### Reads pre-processing

The raw sequencing data generated from the BGI platform were filtered for adapter contamination, low-quality reads, duplicated reads and short reads (length < 35 bp). This filtering produced the high quality ‘final raw data’ with an average sequencing depth of 33.91×.

### Read mapping, SNP calling and annotation

The high quality paired-end sequencing reads (100 bp) from each genotype were mapped to the *P. avium* genome (version 1.0.a1) (https://www.rosaceae.org/species/prunus_avium/genome_v1.0.a1) reference genome using BWA (v0.7.12)^18^. Then the Sequence Alignment/Map (SAM) files were sorted using the Picard software (http://picard.sourceforge.net). GATK genome analysis toolkit (version 4.1.4.1; https://hub.docker.com/r/broadinstitute/gatk/) was employed to call SNPs and InDels across all 21 genotypes simultaneously by using standard hard filtering parameters of HaplotypeCaller and SelectVariant, respectively^19^.

SNP annotation was performed based on the *P. avium* Genome v1.0.a1 using snpEff software^20^, and SNPs were categorized into intergenic upstream or downstream regions and introns or exons. SNPs in coding exons were further classified as synonymous or non-synonymous SNPs. InDels in exons were grouped according to whether they occur a frameshift.

### Genetic diversity and population structure

The 21 genotypes were categorized in 4 groups “Wild”, “Landraces”, “Breeding lines” and “Modern cultivars” (Supplementary File 1). Nucleotide diversity (π) and Tajima’s D of each group and population-divergence (Fixation index, F_ST_) between each group were calculated by using VCFtools (v0.1.14) with a 20 kb sliding window across the *P. avium* Genome v1.0.a1 reference genome.

Population genetic structure was assessed using the following methods. Initially, Nei’s distance was calculated and a neighbor-joining dendrogram of the 21 genotypes was build using StAMPP^21^ and *ape*^22^ R packages. Then, clustering of the 21 genotypes was performed by using the Discriminant Analysis of Principal Components (DAPC) implemented to the R package, Adegenet^23^. The optimal number of clusters was calculated using the Bayesian Information Criterion (BIC) as described by Jombard et al.^23^. Lastly, gene flow among populations was estimated by calculating the F_ST_ for pairwise comparisons for all populations using the StAMPP package in R and hierarchical clustering using the ward.d2 method^24^.

### Structural variant discovery and annotation

BreakDancer (http://breakdancer.sourceforge.net/), SOAPcnv^25^, DELLY^26^ and LUMPY^27^ were used for structure variants (SVs) and copy number variants (CNVs) calling. First, alignment of sequences against the *P. avium* genome was done using bwa-mem pipeline. Then, DELY and LUMPY were used for CNV detection and each.vcf file was validated using “vcf-validator” tool of vcftools^28^. Intergation and filtering of DELLY and LUMPY output was realized using the “methodsMerge” command of the “intansv” package in R with default parameters^29^. The CNV detection was performed by separating DNA sequences into fragments according to the sequencing depth of bases and the alignment results. Then, the *P*-value was calculated for each fragment to estimate its probability to be a CNV. The fragments that passed the criteria (fragment length longer than 2 kb, *p*-value ≤ 0.05, and mean depth <0.5 or >2.0) were identified as CNVs.

### Linkage disequilibrium

Potential linkage disequilibrium was detected by using PLINK. Pairwise *r*^2^ was obtained for all markers within a 0.5 Mb window and data were fitted using a local polynomial regression fitting (LOESS) model^23^ implemented in R (v. 3.3.3)^24^. Background linkage disequilibrium (BLD) was estimated by bootstrapping; 1000 replications were performed, and on each replication, *r*^2^ was calculated among 1000 randomly selected SNPs. The BLD value was chosen as the upper value of the 95% confidence interval of the *r*^2^ distribution.

LD decay was calculated for four groups of populations (a) the entire population of the 21 cultivars, (b) the ‘Breeding line” group, (c) the ‘Landrace’ group and (d) the ‘Modern cultivar’ group.

### Genetic variation on genes related with traits of interest

Based on the report of Sánchez-Pérez et al.^30^, thirty three candidate genes involved in the regulation of flowering time were selected. Additionally, the VCF-BED intersect tool^31^ was employed in order to retrieve the SNPs and InDels variations that were mapped to the 119 annotated disease resistance genes, as well as the 16 pathogenesis-related genes, which play pivotal roles in the defense reactions against pathogens in the reference genome. Genetic variability of the candidate genes was explored across the different accessions and the potential effect of the genetic changes was studied, using snpEff v.4.3^20^, by annotating each SNP, based on their predicted effect on the candidate genes.

## Results and discussion

### Variation in the sweet cherry genome

Genome sequencing of the 21 sweet cherry genotypes yielded 204.26 Gb of high quality clean data with an average sequence depth of 33.91x and effective mapping depth, to the genomic reference sequence of ‘Satonishiki’ cultivar, between 22.21× to 36.62× (Supplementary Fig. 1, Supplementary File 2). These results suggest a well-covered genome with higher sequence and mapping depth compared to similar studies^32,33^.

**Fig. 1:**
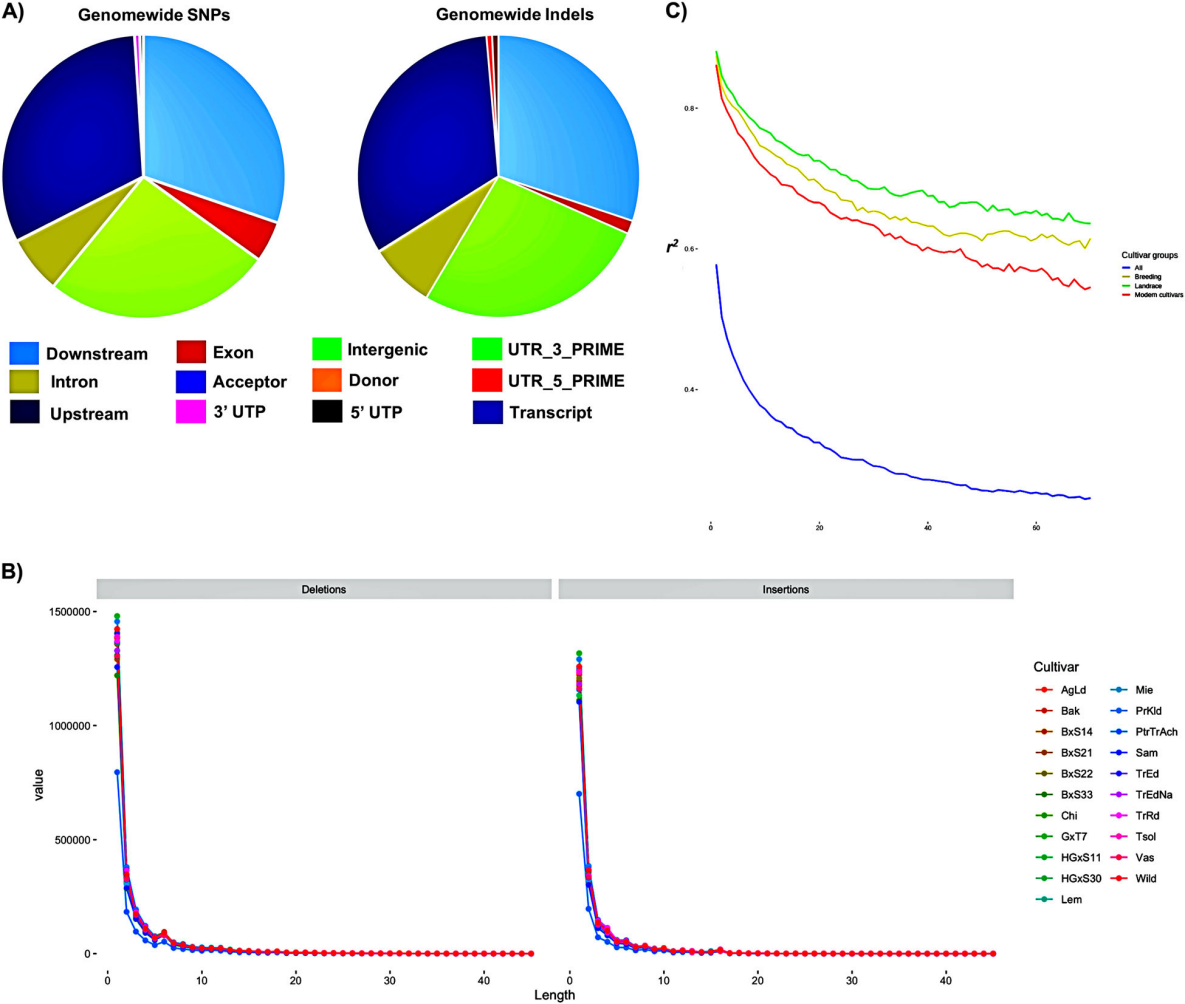
Classification of SNPs and Indels based on genome location: **a** distribution of SNPs and InDels among functional effect classes compared to the proportion of sites in the reference (cv. ‘Satonishiki’) genome, **b** distribution of small insertions and deletions in genomic regions per cultivar, **c** decay of linkage disequilibrium measured as the squared correlation coefficient (*r*^2^) by physical distance in **a** 21 cultivars, **b** the “Landrace” group, **c** the “Modern cultivar” group and **d** the “Breeding line” group

**Fig. 2:**
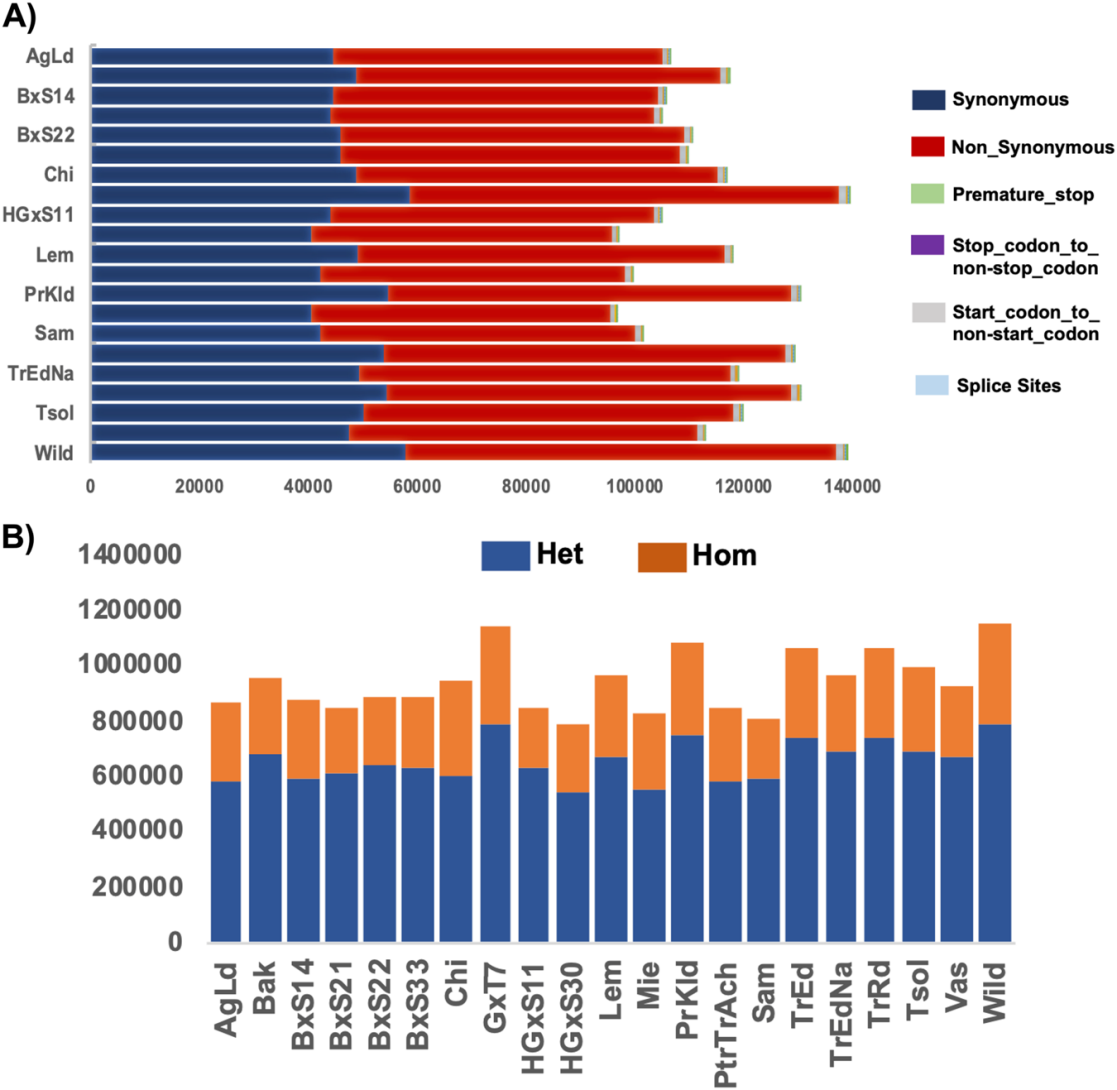
**a** Number of large-effect SNPs in genome of 21 sweet cherry accessions. The *X*-axis displace the sample name, and *Y*-axis shows the number of each kind of large-effect SNP; **b** SNPs and InDels genetic diversity among 21 sweet cherry accessions. Het = Heterozygosity, Hom = Homozygosity

Paired-end sequencing reads were mapped to the genomic reference sequence of *Prunus avium* cv. ‘Satonishiki’^13^ resulting in an average mapping depth of 35.51×. Genome-wide variation including 1,880,922 single-nucleotide polymorphisms (SNPs), 452,544 small insertions or deletions (indels), 5,677 copy number variations (CNVs) and 6607 presence absence variations (PAVs) across 21 sweet cherry genotypes, was identified (Table 1). About 26.15% of the total number of SNPs are located in intergenic regions and 12.13% in coding regions. SNPs in genic regions include 82,081 synonymous, 146,144 non-synonymous substitutions. Moreover, our analysis identified 411,957 intronic variants and 29,730 and 36,647 SNPs in 5′′ and 3′ UTRs, respectively (Fig. 1b). The cultivars ‘Wild’ and ‘Mie’ have the highest and lowest number of SNPs, respectively (Table 1). Due to self-incompatibility of this species, most of the SNPs/InDels are heterozygous and the wild genotype possesses the greatest number of heterozygous SNPs (Fig. 2b). Most of the nucleotide changes can be classified as transitions (11,759,094), with a transition/transversion ratio (Ts/Tv ratio) of 1.4675. Based on the type of change and its predicted effect, 0.36% of the SNPs were predicted to have a high impact (e.g., stop codon gaining, frameshift), 2.34% a moderate (e.g., non-synonymous change, non-disruptive frameshift), and 1.56% a low impact (e.g., synonymous coding/start/stop, start gained) (Fig. 1b). These findings are in accordance with Shirasawa et al.^13^; their values were 0.7%, 6.4% and 4.6% for high, moderate and low impact mutations, respectively. The non-synonymous-to-synonymous substitution ratio (dN/dS) for the SNPs in the coding regions was 1.78. For crops or species that are propagated clonally, synonymous SNPs are outnumbered by non-synonymous SNPs, while the opposite is more commonly met in wild species^34^. However, this value is much higher to the values reported for pigeon pea (1.18^35^), tomato (1.23^34^), peach (1.06^36^), Chinese plum (1.30^37^) and grapevine (1.17^32^). The higher dN frequency in our population of sweet cherry may possibly be the result of the artificial selection pressure imposed by breeding in combination with the vegetative propagation through grafting of domesticated cultivars.

**Table 1:** Genome-wide variations identified in 21 sweet cherry genotypes

	SNPs					Indels						SVs				CNVs		
Cultivar	Total	Intron	Intergenic	Exon	Others	Total	Intron	Intergenic	Exon	Insertions	Deletions	Deletions	Duplications	Inversions	Insertion	Total	Up-regulation	Down-regulation
AgLd	779,132	126,224	530,065	102,093	20,750	150,391	29,081	108,269	7395	73,548	76,843	603	126	4	174	5316	2464	2852
Bak	860,759	141,014	585,353	111,265	23,127	167,309	32,648	120,419	7895	81,772	85,537	750	114	7	187	5731	2338	3393
BxS14	782,724	128,237	531,923	101,533	21,031	153,259	30,000	110,336	7122	75,428	77,831	445	67	7	195	5926	2464	3462
BxS21	758,090	125,235	513,268	98,858	20,729	150,579	29,847	108,020	6971	74,139	76,440	864	144	7	199	5854	2188	3666
BxS22	817,328	132,206	557,723	106,107	21,292	158,071	30,824	114,028	7346	77,491	80,580	618	102	5	168	5742	2243	3499
BxS33	785,432	129,693	532,279	102,226	21,234	151,148	29,833	108,507	7016	73,818	77,330	761	160	4	175	5780	2367	3413
Chi	858,053	141,276	582,002	111,636	23,139	170,673	33,482	122,592	7993	83,807	86,866	754	101	6	173	5519	2317	3202
GxT7	1,044,452	175,356	704,824	135,239	29,033	200,912	40,025	143,832	9321	97,729	103,183	716	102	7	189	5789	2474	3315
HGxS11	770,256	127,502	522,357	99,713	20,684	152,159	30,119	109,329	6964	74,551	77,608	533	91	8	191	5912	2262	3650
HGxS30	712,764	115,899	485,661	92,609	18,595	140,195	27,505	100,968	6555	68,902	71,293	352	66	0	220	5937	2280	3657
Lem	867,613	140,414	591,533	112,580	23,086	170,706	33,051	123,334	7963	83,469	87,237	724	126	6	184	5696	2204	3492
Mie	742,986	120,978	505,660	96,486	19,862	144,774	28,414	104,060	6945	70,823	73,951	472	81	6	207	5540	2461	3079
PrKld	987,103	164,391	668,104	127,691	26,917	194,583	38,355	139,897	8979	95,653	98,930	592	89	7	181	5888	2309	3579
PtrTrAch	728,595	117,664	495,279	95,934	19,718	130,819	25,617	93,720	6424	63,460	67,359	48	6	1	168	4834	2291	2543
Sam	730,523	116,319	497,476	97,182	19,546	139,069	27,023	99,845	6906	68,067	71,002	425	85	5	205	5244	2409	2835
TrEd	951,935	158,497	644,758	123,229	25,451	187,287	36,883	134,658	8727	91,162	96,125	759	121	4	224	5778	2262	3516
TrEdNa	866,018	140,120	590,447	112,897	22,554	168,243	32,501	121,520	7946	82,390	85,853	578	101	3	193	5715	2331	3384
TrRd	953,161	158,303	646,175	123,228	25,455	187,927	37,193	134,937	8707	91,931	95,996	1055	159	8	243	5827	2243	3584
Tsol	888,949	146,417	603,865	114,852	23,815	172,035	33,847	123,503	8161	83,877	88,158	831	143	5	191	5664	2381	3283
Vas	828,192	137,285	562,200	107,493	21,214	163,155	32,332	116,989	7656	79,836	83,319	676	120	3	216	5963	2328	3635
Wild	1,050,518	174,758	712,584	135,030	28,146	201,612	39,551	145,246	9315	97,512	104,100	758	103	2	167	5560	2344	3216
All genotypes	1,880,922	318,805	1,264,593	226,969	133,5148	427,160	85,475	307,160	17,410	80,452	84,073	634	105	5	192	5677	2331	3345

We produced a unified catalog of SVs called by at least two of these four bioinformatics tools and these are described in Table 1. This catalog is comprised by 634 deletions, 192 insertions, 2312 duplications, inversions, or translocations (intra- or inter-chromosomal) and 5677 copy number variants (average length 5.2 kb). The CNVs ranged from 4834 in cv. ‘Petrokeraso Tragano Achaias’ to 5963 in cv. ‘Vasiliadi’. The distribution and corresponding annotations of PAVs and CNVs have been established (Supplementary Files 3 and 4). Shirasawa et al.^13^ WGRS study on six sweet cherry cultivars, has identified a lower number of sequence variants (1,179,268), SNPs (1,016,866) and insertions/deletions (162,402), indicating higher genome wide variations in the present study, probably due to the larger number of genotypes.

**Fig. 3:**
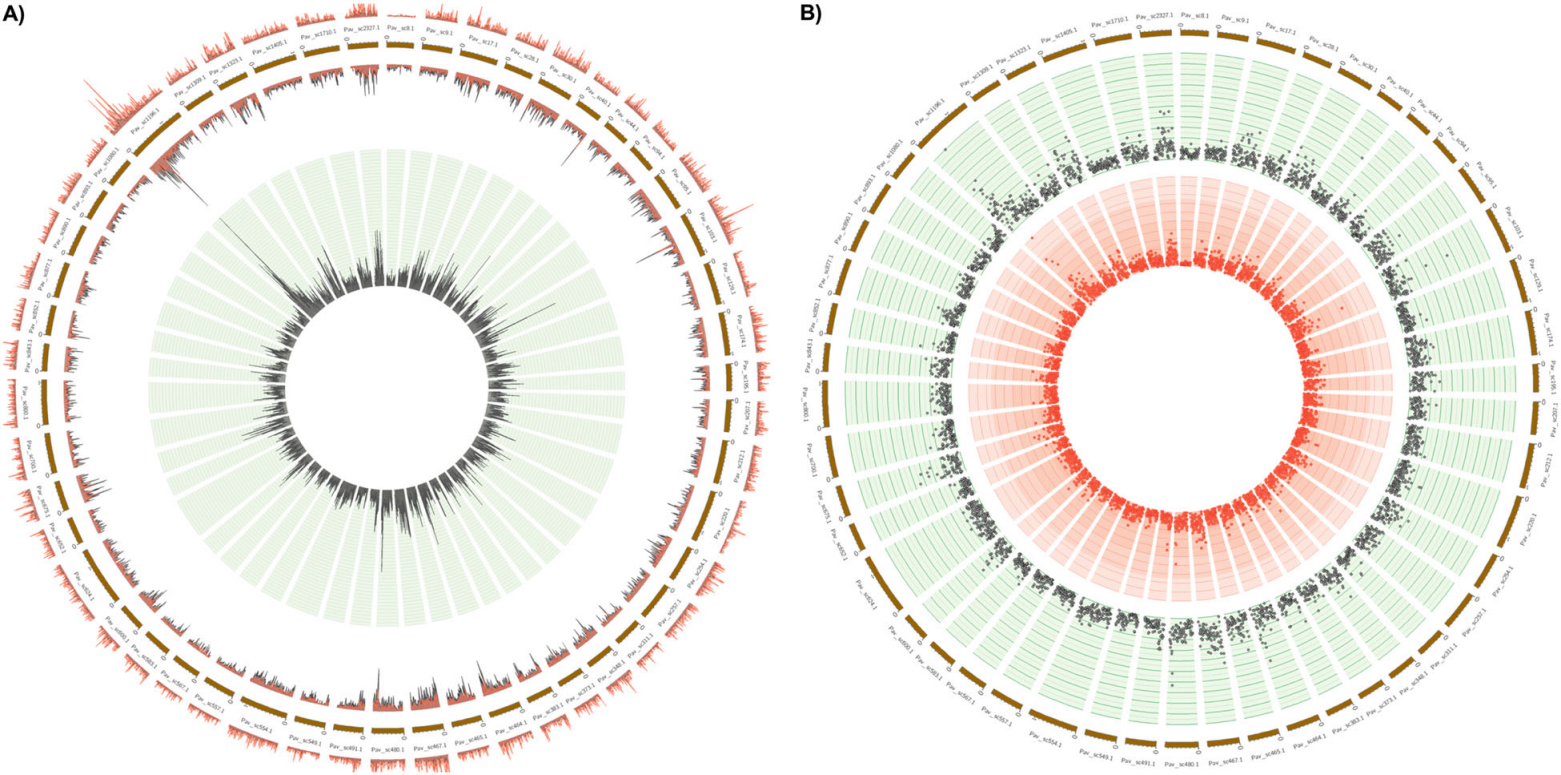
Circos plots of the global distribution of SNPs and InDels in 21 sweet cherry accessions. **a** Outer circle: the distribution of SNPs in 100 kb window; Inner circle: the distribution of indels in 100 kb window. **b** Central circle: the distribution of SNPs and InDels in 100 kb window; outermost circle: the distribution of InDels in 100 kb window; Inward-facing circle: the distribution of SNPs in 100 kb window

Regarding InDels, a total of 427,160 variants were identified, of which 204,376 were insertions and 222,784 were deletions. Among those with potential functional consequences, 1.52% were located within gene exons and 0.2% in splice site regions, while 1.37% of Indels were located in 5′- and 3′- UTR regions. The vast majority of the Indels was located upstream or downstream of genes and in intergenic regions. The size of insertions ranged from 1–29 nucleotides and deletions were in the range of 1–44 nucleotides in length. However, most of the insertions and deletions (25.2%) were of a single nucleotide only. Di- and tri-nucleotide insertions and deletions accounted for 11.02% and 7.4% of the total InDels, respectively (Fig. 1b and Supplementary File 5). Similar results have been observed by Varshney et al.^38^ in 429 chickpea accessions. Moreover, among larger InDels of ≥4-nt, the total number of deletions was slightly higher than insertions (Fig. 1b). Similar to the SNP analysis, the “Wild” genotype demonstrated the greatest number and ‘Mieza’ the lowest number of InDels (Fig. 1a); wild genotype clearly presents the most diverse germplasm reservoir (Fig. 3a, b).

We further analyzed the distribution of so called large-effect SNPs, which may potentially disable gene functions. It was found that within the 3187 SNPs in codon premature termination, 893 SNPs disrupt splicing donor or acceptor sites of the genome, 4211 SNPs are related to alteration of initiation methionine residues, and 571 SNPs replace terminators with curtain amino acid residues that lead to longer ORFs (Fig. 2a).

### Linkage disequilibrium (LD) analysis

Detailed understanding of the linkage disequilibrium in a population of cultivars is crucial when considering the application of association genetics or GWAS in a species. LD is measured as the squared correlation coefficient (*r*^2^) between SNPs decays to 50% of its maximum at 5 kb and 90% of its maximum at 55 kb (Fig. 1c). The majority of SNP pairs with *r*^2^ in near complete disequilibrium (>0.8) are found at physical distances less than 10 kb (Supplementary Fig. 5). This relatively rapid decay of LD suggests that genome-wide association studies (GWAS) form a potential tool applicable for sweet cherry that will enable high-resolution mapping of genes associated with traits of agricultural significance. In comparison to pre-defined groups, we also found that LD decayed rapidly to an average *r*^2^ of 0.8 within 20 kb for both modern cultivars and breeding lines (Fig. 1c). Moreover, the LD decay rate was faster in the modern cultivars than in the landraces suggesting a higher frequency of genetic recombination in the modern cultivars (Fig. 1c).

In *Prunus*, whole-genome diversity has been exploited using both SNPs and whole-genome resequencing data with a minor, or major, depth coverage^39^. Genome-wide data has identified fast LD decay in apricot, spanning <100 bp^40^. Similarly, LD decays fast in ornamental *P. mume* accessions (*r*^2^ ≤ 0.2 at 50 kb to few hundreds of base-pairs depending on the subgroup^37^ and moderately in *P. avium* landraces and cultivars (*r*^2^ ≤ 0.2 at 100 kb^4^). The lower LD extensions oberved in sweet cherry (*P. avium*) compared to the LD observed in peach (*P. persica*) (*r*^2^ ≤ 0.2 between 0.8 and 1.4 Mb depending on the population^41^, are possibly related to the self-incompatibility system that was previously described in sweet cherry^4^.

### Population structure of sweet cherries

**Fig. 4:**
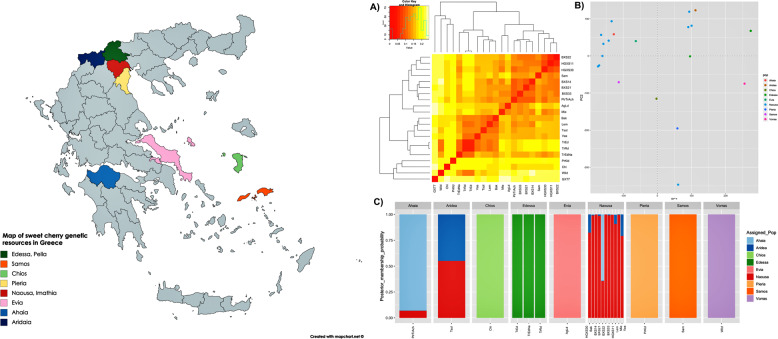
Population structure in cultivated sweet cherries. **a** Heat map depicting genomic relationships between individual sweet cherry accessions lines. Individuals are across the *X*- and *Y*-axis, with each square indicating the genomic relationship between the two respective individuals. The magnitude of the relationship is indicated by the color range (one being the relationship between an individual and itself, and 0 being the mean of the data set). **b** Principal component analysis (PCA) plot of the first two components of 21 cultivars using whole genome SNP data. PC1 and 2 axes account for 21.4 and 6.59% of the variation, respectively; **c** DAPC scatterplots and membership probabilities for SNP data. The scatterplots show the first two principal components of the DAPC. Geographic samples are represented in different colors with individuals shown as dots. Membership probabilities (in bar plots), interpreted as proximities of individuals to different clusters, show clear-cut separation of genetic groups for the SNP data

The population structure of the Greek sweet cherry germplasm was studied by employing the whole genome sequencing data. The hierarchical clustering and neighbor-joining (NJ) tree methods, group the genotypes according to their genealogy (Fig. 4a and Supplementary Fig. 2). Similarly to the NJ tree, PCA has separated the Greek genotypes into three main groups (Fig. 4b). Additionally, TrRd and TrEd are clustered, but their (short) distance does indicate some level of genetic divergence among them. Ganopoulos et al.^2^ reported similar clustering using SSR markers. The discriminant analysis of principal components (DAPC) with SNPs revealed a clear separation of distinct genetic clusters. Membership probabilities, interpreted as proximities of individuals to different clusters^23^, showed that genome-wide SNP markers achieved unambiguous separation of all groups (Fig. 4c).

DAPC results presented a similar picture to that portrayed by PCA, but at a greater detail. Our germplasm collection appears to be separated into two clusters (Supplementary Fig. 3A). The optimal number of two clusters was also indicated by the Bayesian Information Criterion (BIC) statistic (*K* = 2) based on the Discriminant Analysis of Principal Components (DAPC) (Supplementary Fig. 3B). Cluster 1 (green) comprised of 11 accessions, most of them originating from the area of Naousa and four other accessions originating from the areas of Aridea, Chios island, Evia and Samos island. Cluster 2 (orange) includes a diverse group of accessions composed of 10 sweet cherry cultivars, mostly from the area of Edessa, and one wild cherry genotype.

We studied whether the groups defined a priori, represent statistically significant subpopulations by pairwise comparison of two measures of differentiation; Fst and Nei’s standard genetic distance (Dst). The highest genetic distance and Fst values were observed between the breeding lines group and the wild species (Table 2). On the contrary, the minimum genetic distance was determined between landraces and modern cultivars suggesting a narrow genetic background of currently cultivated sweet cherry cultivars.

**Table 2:** Genetic distances between the four predefined groups

Predefined groups	Landraces	Modern cultivars	Breeding lines	Wild
Landraces	0			
Modern cultivars	0.018	0		
Breeding lines	0.029	0.094	0	
Wild	0.037	0.13	0.2	0

Pairwise estimates of Nei’s standard genetic distance (Dst) between the predefined groups. Color scale: Red = 0 to Blue = 1.

**Fig. 5:**
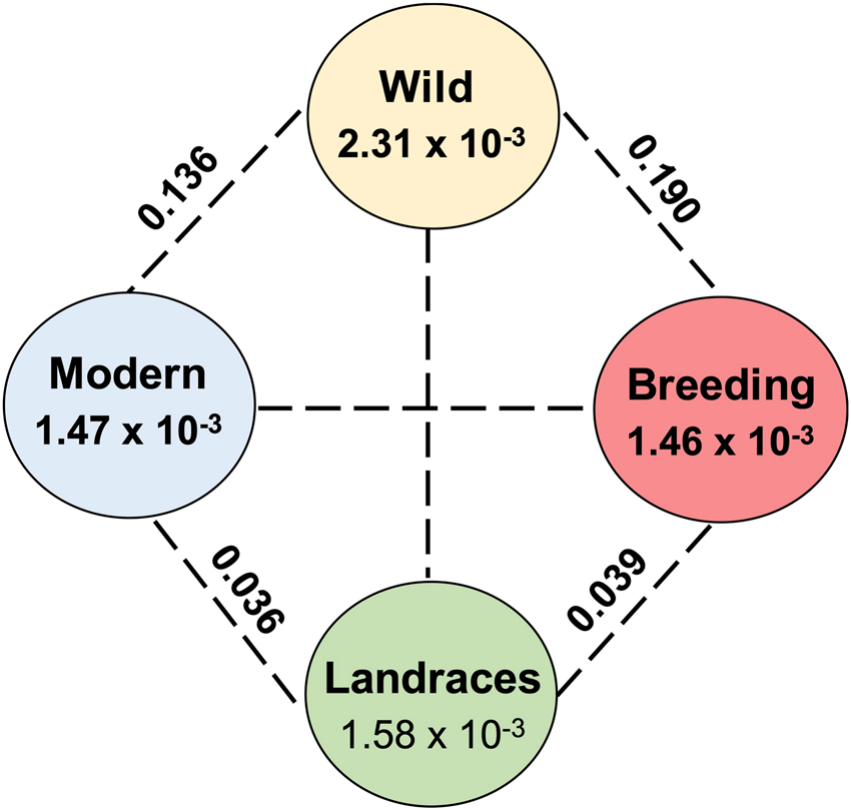
Nucleotide diversity (*π*) and population divergence (Fst) across the four groups. Values between pairs (dashed-line) indicate population divergence (Fst) and value in each circle represents nucleotide diversity (*π*) for the group

The wild progenitor of Greek cultivated sweet cherry cultivars, showed the highest nucleotide diversity (2.31 × 10^−3^) among the four predefined groups (Fig. 5). A very narrow domestication bottleneck was observed between the wild progenitor and landraces (π-wild/π-landraces = 1.46). This bottleneck is much weaker than the one observed in peach^36^ (π-wild/π-landraces = 2.92), but still higher than other fruit-tree species such as grape^42^ and apple^43^ that lack domestication bottlenecks, indicating an effect of the artificial selection on the sweet cherry genomes.

The “modern” (*π* = 1.47 × 10^−3^) and “breeding” (*π* = 1.46 × 10^−3^) cultivars share the same level of genetic diversity, suggesting a similar genetic background of the current cultivated sweet cherry cultivars, that retained the genetic diversity during their traits’ improvement. However, the positive Tajima’s D values (Supplementary File 7) and the limited genetic diversity and differentiation (Fst = 0.09) between the “modern” and “breeding cultivars” is a major constrain for further trait improvement, suggesting that future breeding programs need to introgress material from wild genotypes.

### Variation in genes involved in flowering time and dormancy

**Fig. 6:**
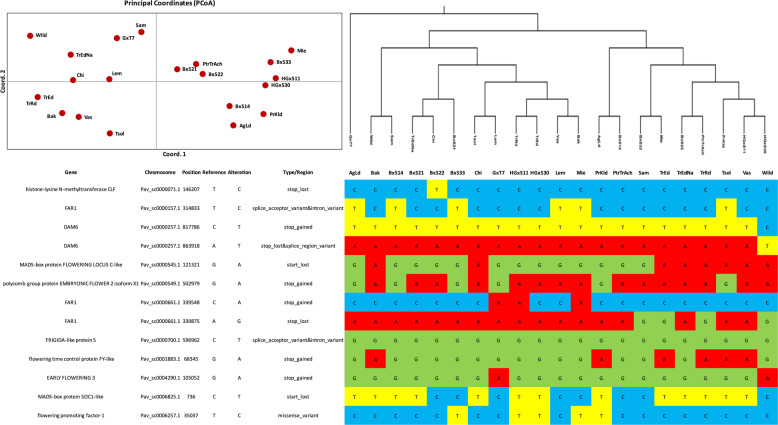
Twenty-four SNPs with high impact alterations identified in genes involved in flowering time and dormancy

A set of 13 candidate genes were selected, based on previous QTL studies, that are involved in flowering time and bud dormancy among various sweet cherry cultivars and other well-studied model plants such as *Arabidopsis thaliana*, i.e., Histone-lysine N-methyltransferase CLF (CLF), the Polycomb group (PcG) protein, the Embryonic Flower gene (EMF2), FLOWERING LOCUS C (FLC), MADS‐box transcription factor SUPPRESSOR OF OVEREXPRESSION OF CO 1 (SOC1), FRIGIDA (FRI) and Far-red-impaired responsive protein (FAR1)^39,44^. The dataset of SNPs found in each gene is summarized in Supplementary File 6 and the mutations in the 5´ or 3´UTR, exons and introns with high impact to the function, such as stop codon gain/loss, of the 13 candidate genes are reported on Fig. 6.

The majority of knowledge on the genetic basis of flowering time is obtained from studies on *Arabidopsis thaliana* (see ref. ^30^ and references therein). More than 60 genes have been identified to control flowering time in *Arabidopsis*^45^. Among them, SNPs present in genes including CONSTANS (CO), FLOWERING LOCUS C (FLC), VERNALIZATION INSENSITIVE 3, PHYTOCHROME D, GIBBERELLIN, etc. or coding region deletions as in FRIGIDA gene, result in significantly different phenotypes for flowering time. Dominant alleles of FRI generate late flowering phenotypes while FRI mutants are early flowering^46–48^ identified ParSOC1 as a candidate that could be involved in the regulation of dormancy break of vegetative shoots in apricot. Loss-of-function mutations in the EMF genes result in direct flowering in *Arabidopsis*, bypassing vegetative shoot growth^49^.

In the present study, no high-impact mutations were found in the flowering time T genes (FT), but some moderate variants in these genes (missense_variant; p.Ser175Tyr) were detected (Supplementary File 6).

Other high impact mutations affected genes associated with bud dormancy. A stop gained mutation specific of the only wild cherry genotype used, was found in the dormancy-associated MADS-box (DAM6) (Pavsc0000257.1). Dormancy-associated MADS-box (DAM) gene expression has been implicated in the establishment and maintenance of endodormancy. DAM6 genes are upregulated during growth cessation and downregulated by cold exposure during winter^39^. DNA methylations and small interfering RNAs are involved in the silencing of the sweet cherry PavMADS1 during cold accumulation and dormancy release^50^ with silencing of PavMADS1 and PavMADS2 coinciding with an increase in *Flowering Locus T* expression during dormancy in *P. avium*.

According to the results obtained from this analysis, some cultivars clustered according to their flowering time (Fig. 6). For instance all of the late-flowering cultivars clustered together; while similar clustering was observed for most of the early-flowering cultivars. The subset of early-flowering cultivars that are clustered with those of late-flowering possibly, suggests that additional genes have an important impact on the flowering time of these sweet cherry cultivars. However, the observed genetic variation in flowering time related genes among the Greek sweet-cherry cultivars could be a valuable source of genetic markers for future breeding programs.

### Variation in genes involved in defense reactions against pathogens

In this study, we selected a set of 119 defense-related genes (*RPM1*, *RPP13*, *RGA2* homologs) which were previously predicted and being functionally annotated as disease resistance genes in the sweet cherry genome according to Shirasawa et al.^13^. These genes are usually clustered in close physical mapping and underlying QTLs have been previously described as involved in defense reactions against numerous plant pathogens^51^.

**Fig. 7:**
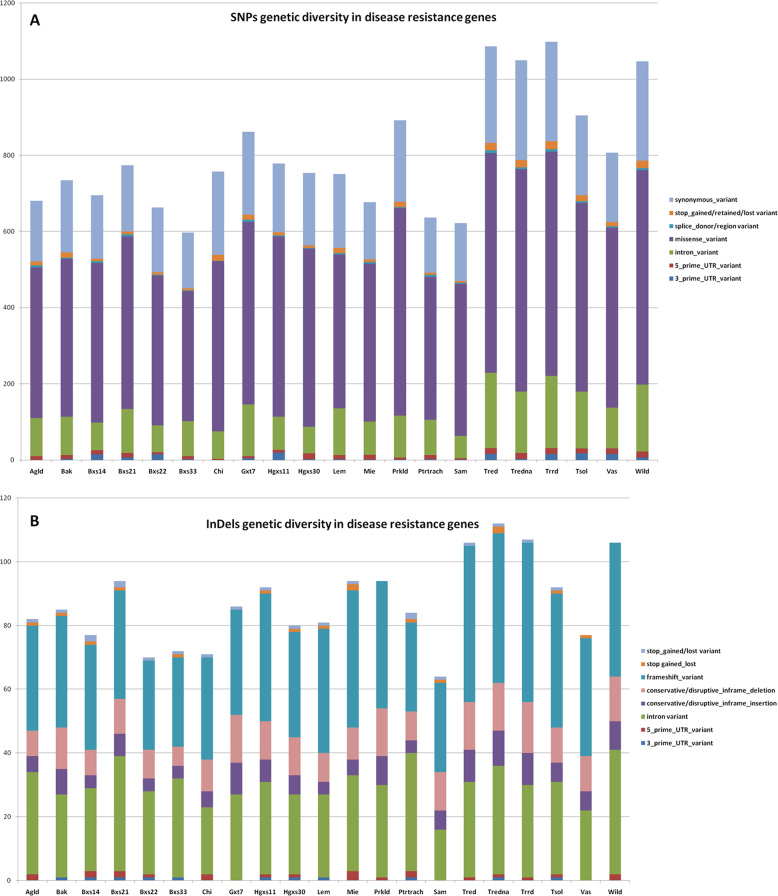
Distribution of SNPs (**a**) and InDels (**b**) based on the variants effects in the disease resistance genes among the 21 sweet cherry accessions. The X-axis displaces the sample name, and Y-axis shows the number of each kind of effect variant (mutation)

The SNPs and InDels variations observed according to our WGRS across the 21 sweet cherry accessions in each disease resistance gene are summarized in Supplementary Files 8 and 9. The total number of variants were 2468 and they were mapped on 107 *R* genes (almost 90% of the genes tested). The SNPs distribution (2241 in total) among the 21 genotypes and according to their annotation effect is depicted in Fig. 7a. The majority of them (1188 SNPs) was heterozygous and were missense variants. Forty-four *R* NBS-LRR genes, have stop-codon gain and loss SNP mutations in the coding sequences (CDS) with high impact (Supplementary File 8). A total number of 227 InDels and their effect were identified (Fig. 7b; Supplementary File 10). The insertions and deletions length were up to 41 and 91 bp, respectively. More than 42% (97 InDels) were of high impact referring mainly mutations of frameshift variant annotations effects. The genotypes “Trrd”, “Tred”, “Tredna” and “Wild” demonstrated the largest number of high impact SNPs and InDels variations, among the 21 sweet cherry genotypes (Fig. 7).

**Fig. 8:**
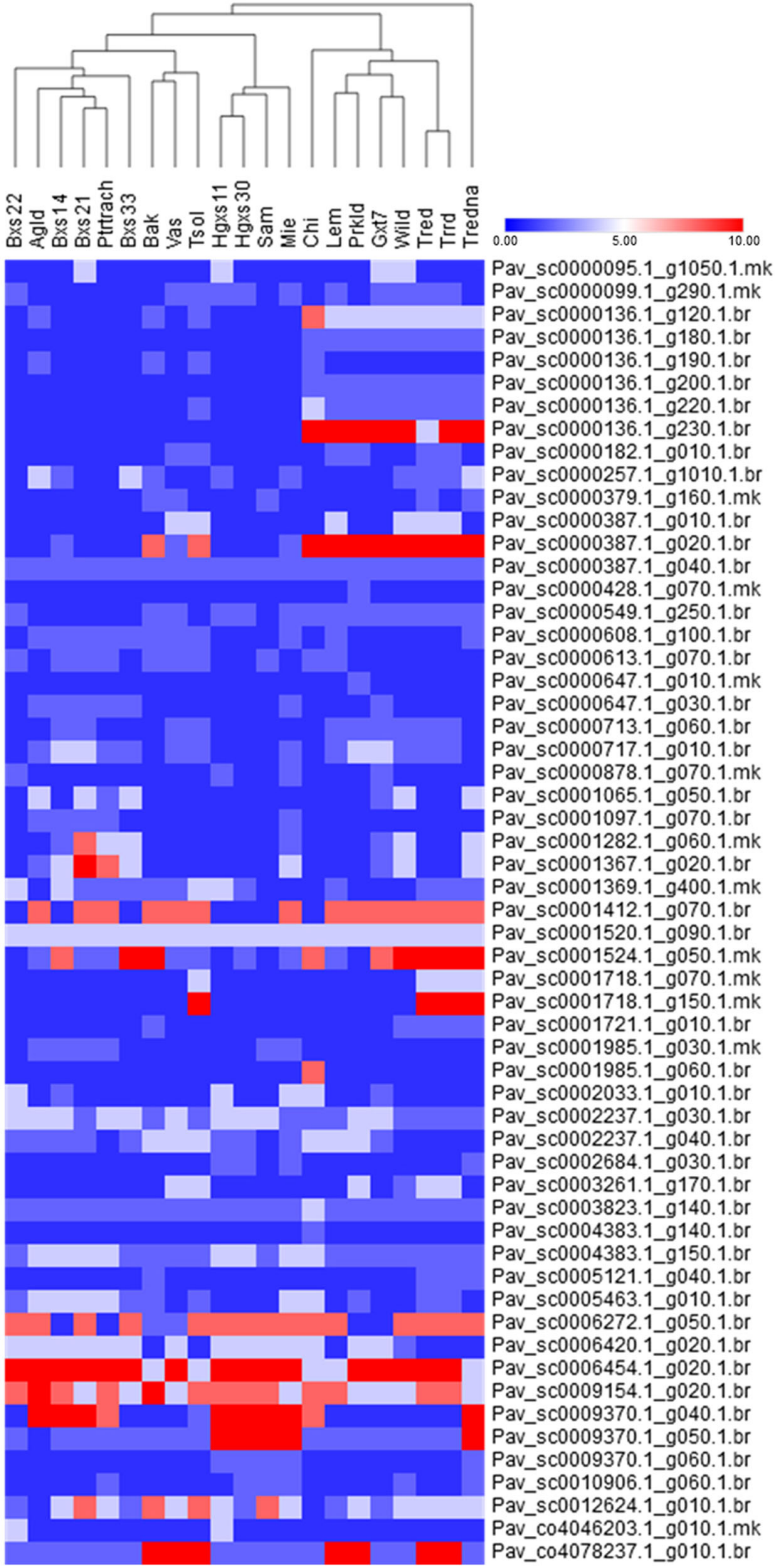
Hierarchical clustering heatmap depicting genomic relationships between individual sweet cherry accessions for the total number of SNPs and InDels with high impact mutations identified and mapped in 57 *NLR* genes involved in defense reactions against pathogens. Sweet cherry accessions and *NLR* genes are across the X- axis and Y-axis, respectively. The magnitude and the color range of the relationship are indicated by the total number of variations across each sweet cherry accession for each *NLS* gene

In order to have a more deciphering view, the 21 sweet cherry genotypes were hierarchically clustered by using all the high impact mutations, both SNPs and InDels, in the 57 *NLR* genes (Fig. 8). The “Tredna” cultivar was found to have the most abundant high impact variants. The “Wild” and “Gxt7” genotypes were clustered together with “Tred” and “Trrd”. Among the 57 genes which contributed mostly in the variants with high impact (Pav_sc0000136.1_g230.1.br, Pav_sc0000387.1_g020.1.br, Pav_sc0006454.1_g020.1.br), the first two contain NB-ARC domains, whereas Pav_sc0006454.1_g020.1.br contains two copies of LRRs of type LRR_4. These genes are homologs of *RPM1* which is a quite dynamical polymorphic locus in *Arabidopsis*^52^. More recently, an evolutionary analysis in sweet cherry genome revealed also that diversifying episodes acting on the NB-ARC domains of Resistance Gene Analogs (RGAs) occurred putatively affecting their ligand-binding specificities through positive selection^3^. Thus, the results of this WGRS analysis, that showed variation in highly abundant SNPs and InDels occuring in NLR genes confirm that these *NLR* receptors are prone to variation among the sweet cherry accessions. All these findings allow us to assign these NSB-LRR proteins as the foremost surveillance mechanism against rapidly evolving pathogens, and providing breeders with effective genomics tools to speed-up the development of sweet cherry varieties with more durable resistance.

We also selected the functionally annotated PATHOGENESIS-RELATED (PR) proteins which encode plant species-specific genes induced in response to infection with fungi, bacteria or viruses in order to estimate the variation upon them across the 21 accessions. These genes have been associated with basic-scale defenses through coordinated interactions within signaling pathways leading at the establishment of systemic acquired resistance (SAR) as well as induced systemic resistance (ISR)^53^. Most of the *PR* genes in sweet cherry genome belong to PR-1 and PR-4 families.

Our results showed that 65 SNPs and 20 InDels variations was observed among the *PR* genes across the sweet cherry accessions (Supplementary Files 10 and 11). These variations were found to disperse across nine out of the 16 *PR* genes in the genome. The majority of them (25 and 15, respectivelly) were found to map in two genes (Pav_sc0000029.1_g200.1.mk and Pav_sc0000030.1_g1270.1.mk spanning scaffolds Pav_sc0000029.1 and Pav_sc0000030.1, respectively).

The majority of these 65 SNPs were intron variants (46 in their number), eight were 3′ and 5′ prime UTR variants and 11 were synonymous, missense and splice-region variants (Supplementary Files 10 and 11). The highest number of variations were found in Lem (58), Trrd (56), while the lowest in Hgxs11 (25) genome accession. The majority of InDels mutations were of intron variant effect with no high impact.

Overall, as it was expected, we found fewer variations among the 21 sweet cherry accessions for *PR* genes in comparison with the disease resistance *NLR* receptors (NBS-LRR-containing genes), as *PR* genes comprise the basal defense and they are more conservative in structure, under rather purifying selection and less abundant in numbers across plant genomes. Therefore, our results indicate that *NLR* genes are promising resources for breeding broad-spectrum resistance as it was previously also mentioned^54,55^.

## Conclusion

This is the first report of whole genome genetic variation characterization between sweet cherry cultivars. By WGRS we have captured the majority of genetic variation (>95%) of Greece’s cultivated germplasm. A high degree of heterozygosity and potentially functional variation was revealed, as indicated by the high nonsynonymous-to-synonymous substitution ratio. Most of the genotypes were clustered according to their geographic region of origin, with some exceptions, which indicates potential movement of the germplasm across regions.

The evaluation at whole genome level of cultivated and wild germplasm is important for the identification of allelic variations with phenotypic effects. We have discovered numerous high impact allelic variants on flowering and diseases resistance genes. Further characterization of the precise impact of such variants on flowering time and defense reactions against pathogens will enable their implementation in molecular breeding programs to fine tune flowering, maturity time and diseases resistance in cherry cultivars. Hence, our study has established the foundation for further large scale characterization of sweet cherry germplasm, aiming at enabling breeders to use diverse germplasm and allelic variants towards developing improved cherry varieties with increased productivity.

## Supplementary information

Additional information R1.

Supplementary Figure 2

Supplementary Figure 3

Supplementary Figure 4

Supplementary Figure 1

Supplementary File 1

Dataset 1

Supplementary File 3

Supplementary File 4

Supplementary File 5

Supplementary File 6

Supplementary File 7

Supplementary File 8

Supplementary File 9

Supplementary File 10

Supplementary File 11

## Data Availability

All of the raw reads of the sweet cherry accessions generated in this study have been deposited in the public database of National Center of Biotechnology Information under SUB6334624.
