# Supplementary material for: Whole genome re-sequencing of sweet cherry (Prunus avium L.) yields insights into genomic diversity of a fruit species
Source: Hortic Res. 2020 May 1;7:60. doi: 10.1038/s41438-020-0281-9 (PMC7193578; doi:10.1038/s41438-020-0281-9)
Supplement: Supplementary file 1 — Additional information R1. [file 41438_2020_281_MOESM1_ESM.docx]

**Whole genome re-sequencing of sweet cherry (*Prunus avium* L.) yields insights into genomic diversity of a fruit species**

Aliki Xanthopoulou, Maria Manioudaki, Christos Bazakos, Christos Kissoudis, Anna-Maria Farsakoglou, Evangelos Karagiannis, Michail Michailidis, Chrysanthi Polychroniadou, Antonios Zambounis, Konstantinos Kazantzis, Athanasios Tsaftaris, Panagiotis Madesis, Filippos Aravanopoulos, Athanassios Molassiotis, Ioannis Ganopoulos

**Supplementary data**

Supplementary Figures 1-5 and Supplementary Files 1-11

**
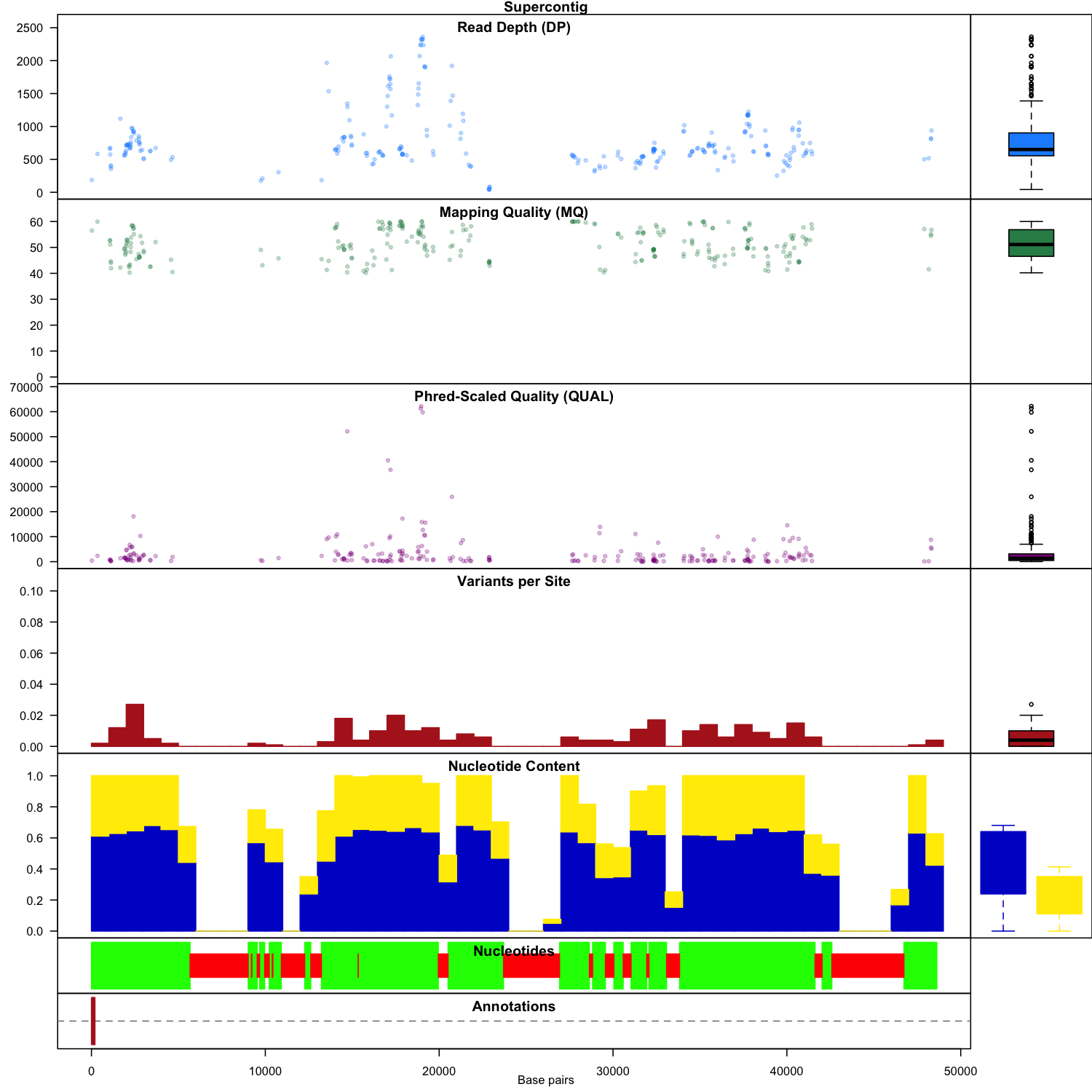
**

**Supplementary Figure 1**

**Supplementary Figure 2**

**Supplementary Figure 3**

**Supplementary Figure 4**

**Supplementary Figures**

Supplementary Figure 1: Chromoqc plot showing raw variant call format (VCF) data for one supercontig in the pinfsc50 data set. The lowest panel represents annotations as red rectangles. Above this is a panel where regions of called nucleotides (A, C, G or T) are represented as green rectangles and ambiguous nucleotides (N) are represented as red, narrower, rectangles. Continuing up the plot is a sliding window analysis of GC content and then one of variant incidence. Above these are three dot plots of phred-scaled quality (QUAL), mapping quality (MQ) and read depth (DP).

Supplementary Figure 2: Population structure in sweet cherries accessions analyzed.

Supplementary Figure 3: A. Posterior membership probabilities for individuals into clusters. B. Selection of number of clusters was based on Bayesian Inference Criterion (BIC), which indicates 2 clusters (green and orange) for data summarization.

Supplementary Figure 4: Summary of Linkage disequilibrium in sweet cherry. Counts of SNP pairs in r^2^ bins plotted as function of the physical distance between each pair

**Supplementary Files**

Supplementary File 1: Detailed information on the sweet cherry accessions in the present study.

Supplementary File 2: Quality of cleaned and filtered data. Mapping rate of each accession.

Supplementary File 3: Structural variation (SV) annotation statistical results.

Supplementary File 4: Copy number variation (CNV) annotation statistical results.

Supplementary File 5: Size in length of insertions and deletions.

Supplementary File 6: Details of SNPs variations of flowering time and dormancy genes across the 21 sweet cherry accessions.

Supplementary File 7: Tajima’s D values estimation of the three predefined groups “Breeding”, “Landraces” and “Modern cultivars”. The “Wild” genotype is not included in this table because Tajima’s D estimation requires a minimum of three genotypes on each group. The positive Tajima's D values signify low levels of both rare and high frequency polymorphisms on each group, suggesting a balancing selection.

Supplementary File 8: Details of SNPs variations of disease resistance genes across the 21 sweet cherry accessions.

Supplementary File 9: Details of InDels variations of disease resistance genes across the 21 sweet cherry accessions.

Supplementary File 10: Details of SNPs variations of pathogenesis-related genes across the 21 sweet cherry accessions.

Supplementary File 11: Details of InDels variations of pathogenesis-related genes across the 21 sweet cherry accessions.
