# Supplementary figures and images for: Whole genome re-sequencing of sweet cherry (Prunus avium L.) yields insights into genomic diversity of a fruit species
Source: Hortic Res. 2020 May 1;7:60. doi: 10.1038/s41438-020-0281-9 (PMC7193578; doi:10.1038/s41438-020-0281-9)

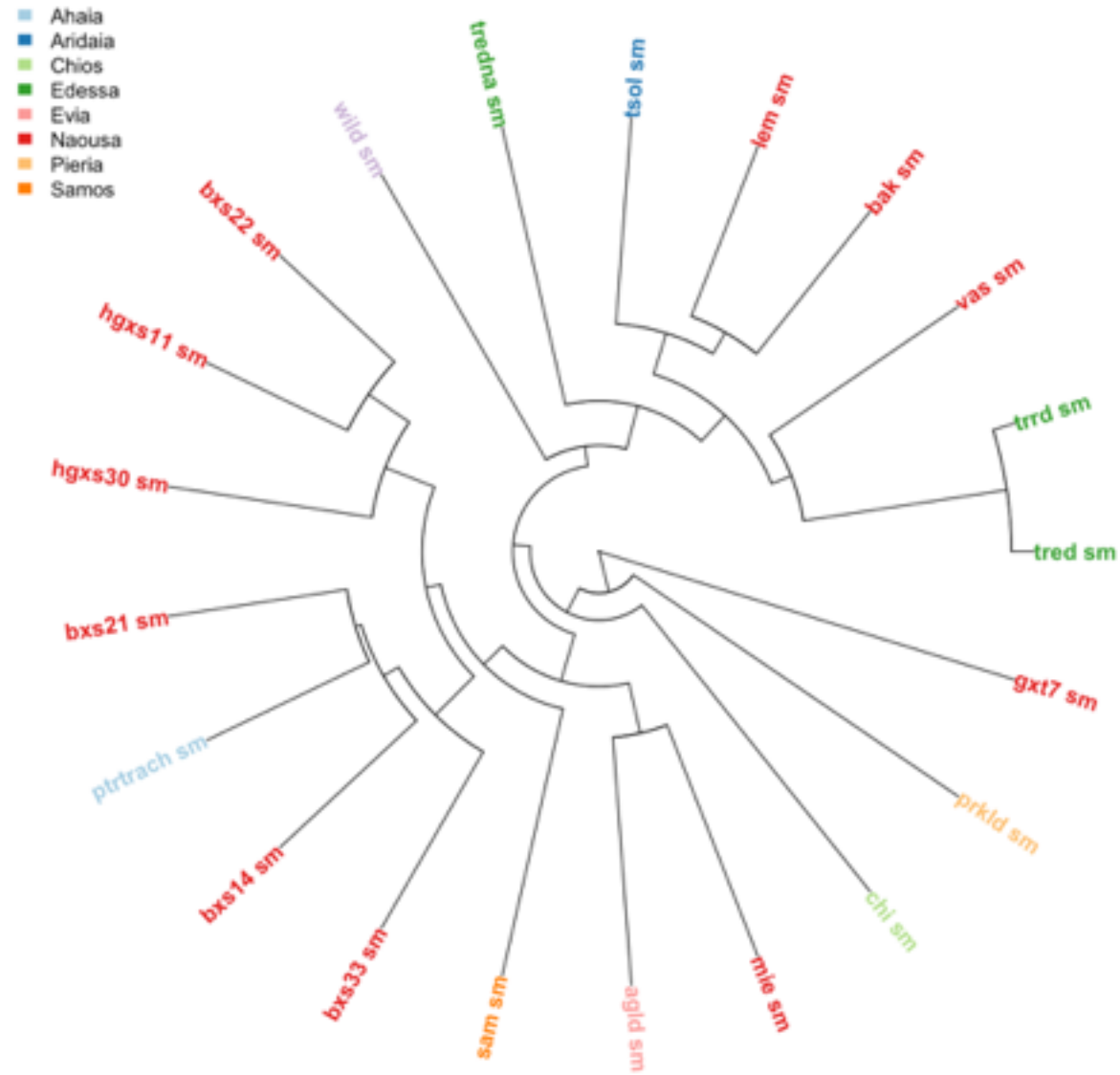

# POPULATION

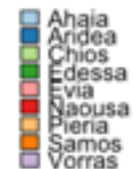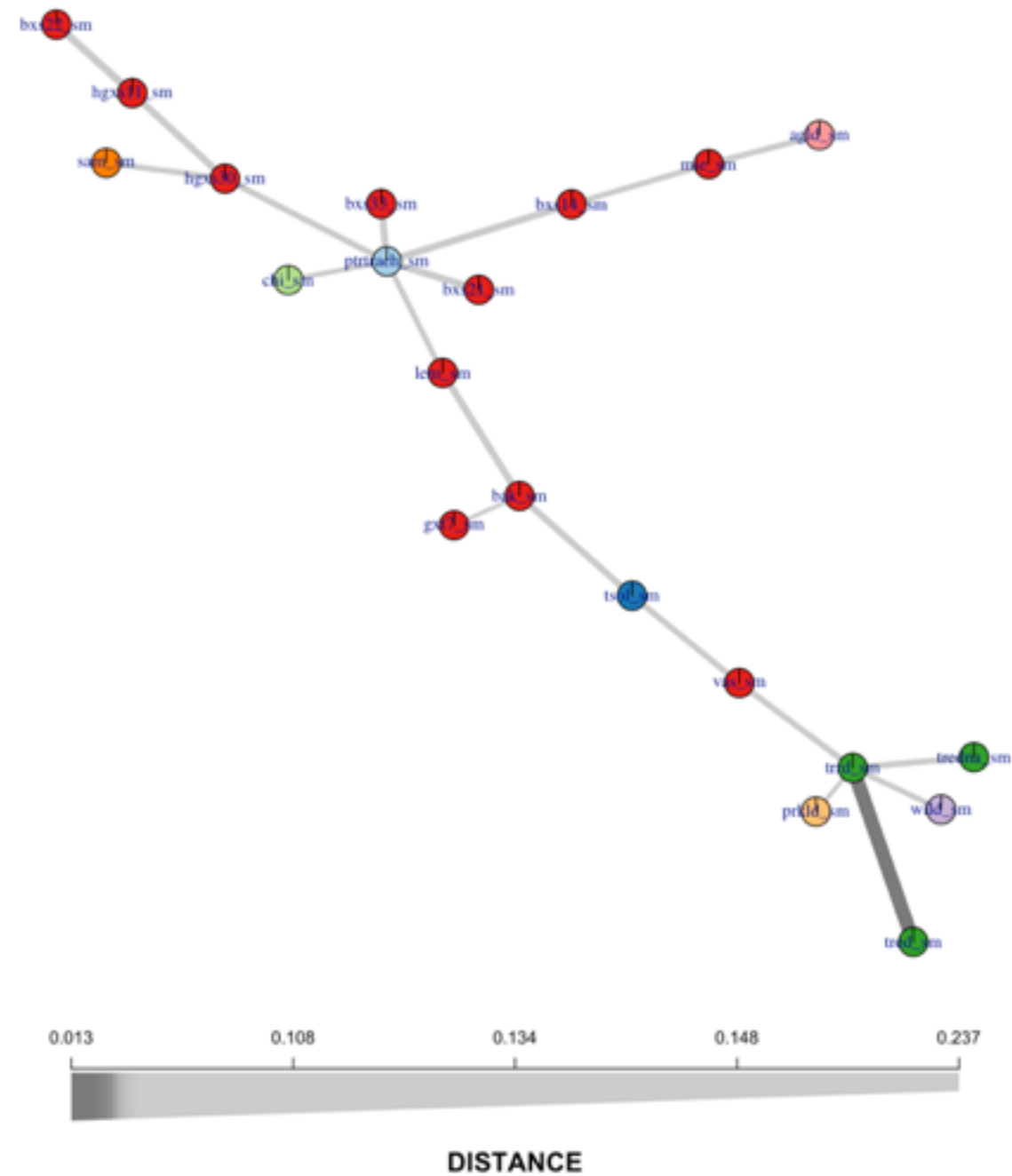

Supplement: Supplementary file 2 — Supplementary Figure 2 [file 41438_2020_281_MOESM2_ESM.pdf]

A)

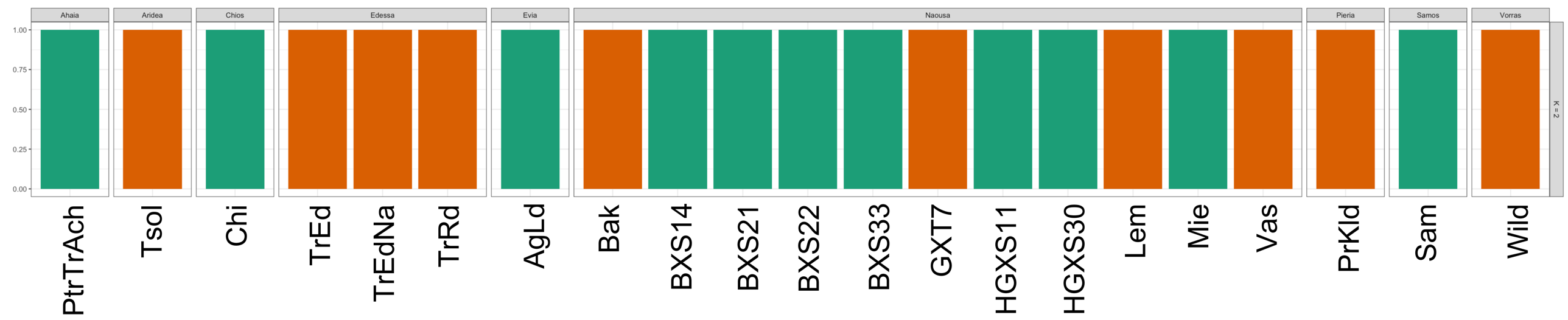

B)

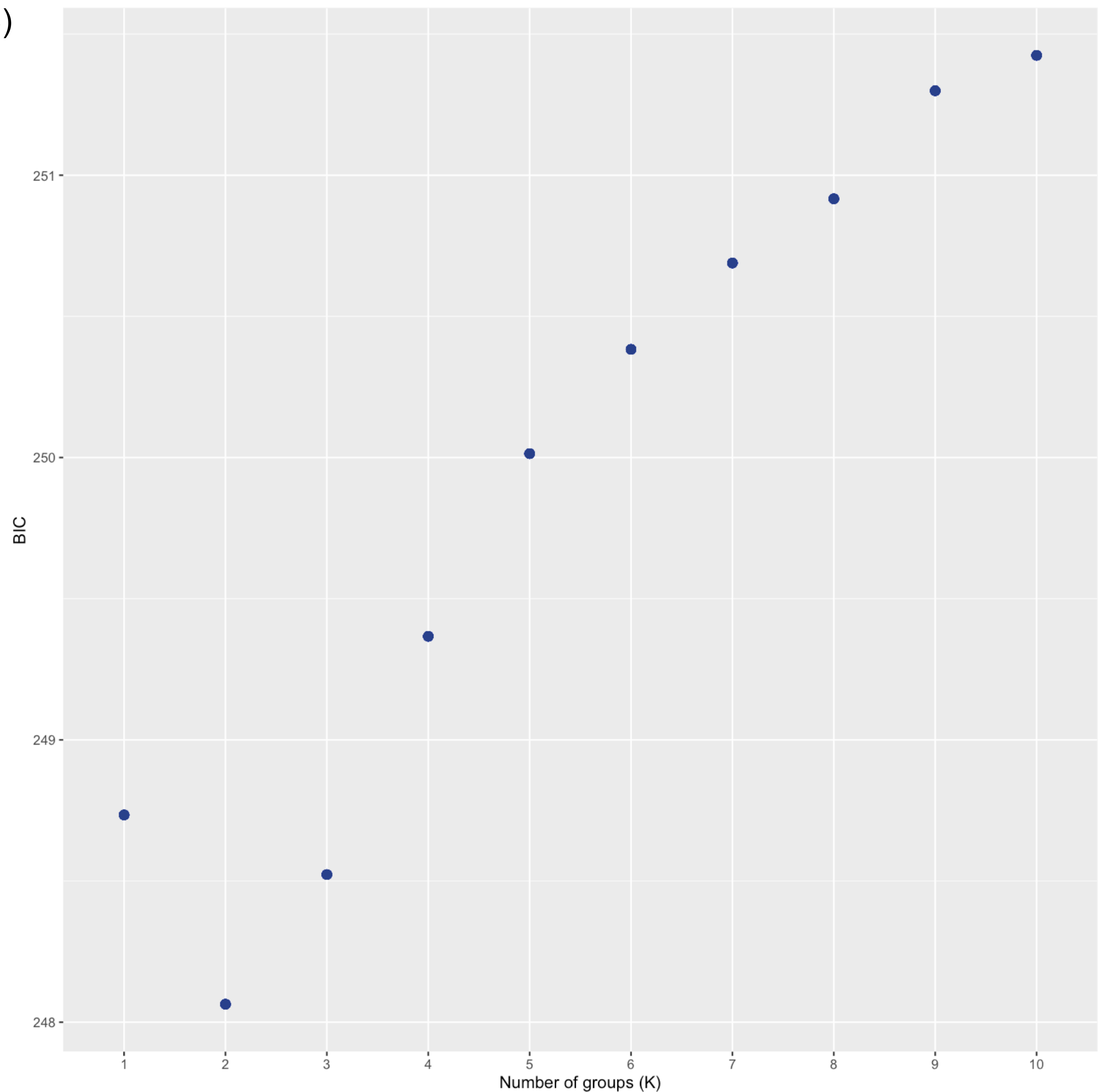

Supplement: Supplementary file 3 — Supplementary Figure 3 [file 41438_2020_281_MOESM3_ESM.pdf]

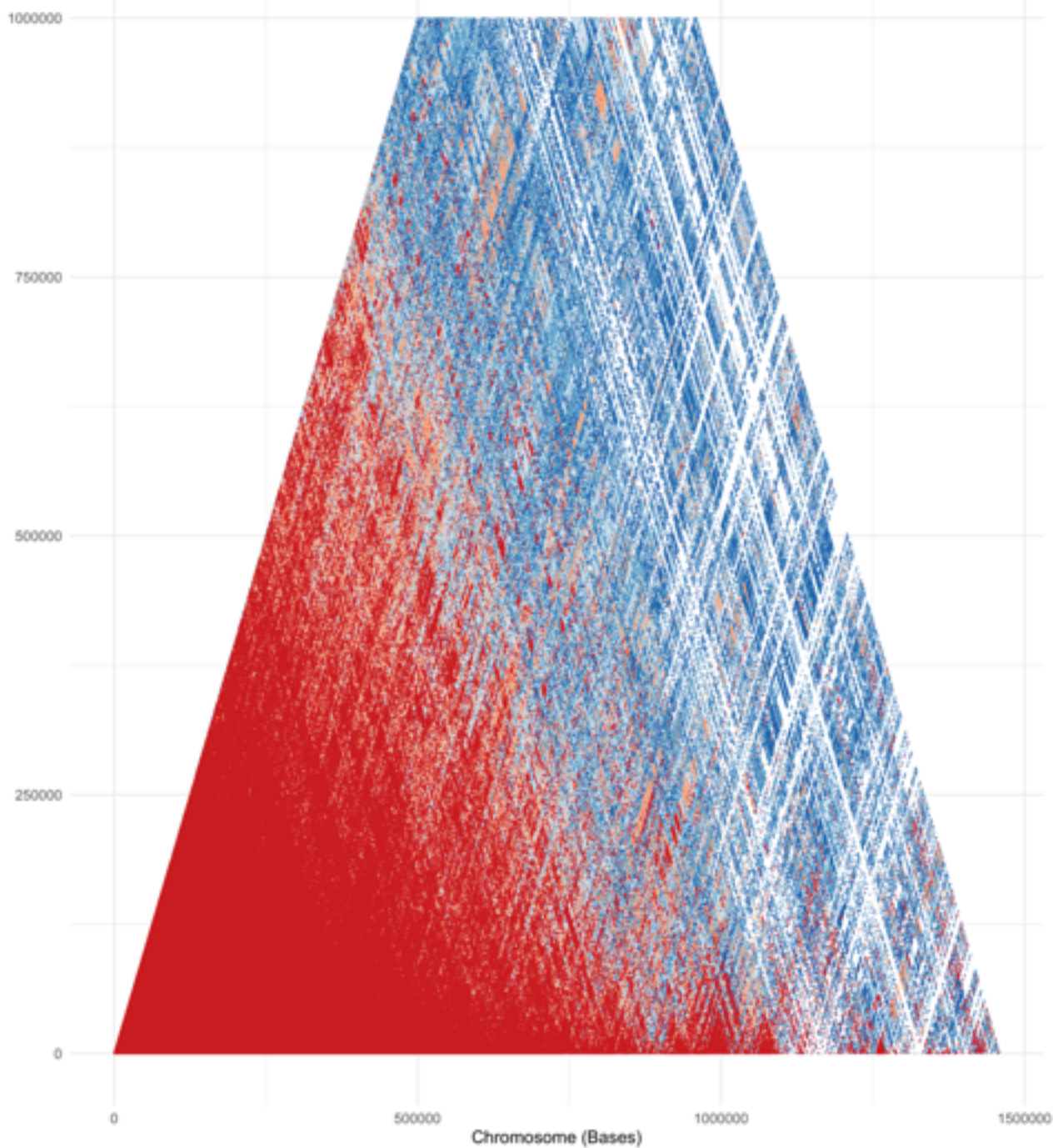

Supplement: Supplementary file 4 — Supplementary Figure 4 [file 41438_2020_281_MOESM4_ESM.pdf]

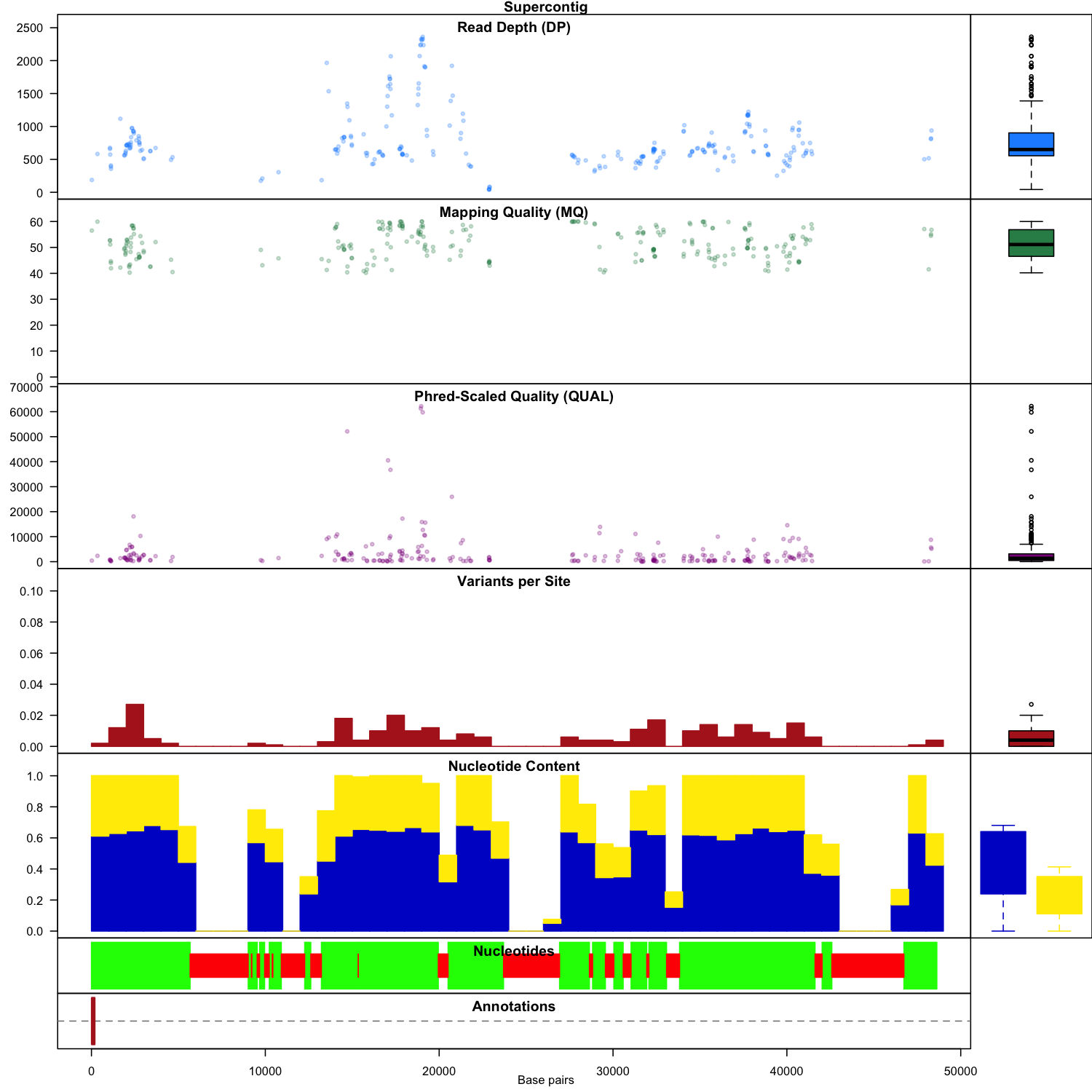

Supplement: Supplementary file 5 — Supplementary Figure 1 [file 41438_2020_281_MOESM5_ESM.png]
